# Reviewer acknowledgement 2012

**DOI:** 10.1186/1880-6805-32-3

**Published:** 2013-03-20

**Authors:** Akira Yasukouchi

**Affiliations:** 1 Department of Physiological Anthropology, Faculty of Design, Kyushu University, Fukuoka, Japan

## Abstract

**Contributing reviewers:**

The editors of Journal of Physiological Anthropology would like to thank all our reviewers who have contributed to the journal in Volume 31 (2012).

## 

Tatsuro Amano

Japan

Sumiko Anno

Japan

Kiyoshi Aoyagi

Japan

Douglas E. Crews

United States of America

Janet Dufek

United States of America

Katsuo Fujiwara

Japan

Yoshiyuki Fukuba

Japan

Yoshiyuki Fukuoka

Japan

Elena Godina

Russian Federation

Hanns-Christian Gunga

Germany

Antonio Gutierrez

United States of America

Hajime Harada

Japan

Shinya Hayasaka

Japan

Shigekazu Higuchi

Japan

Masahiro Horiuchi

Japan

Yuhei Ichimaru

Japan

Kaoru Inoue

Japan

Yoshimitsu Inoue

Japan

Keita Ishibashi

Japan

Koichi Iwanaga

Japan

Masatoshi Katagiri

Japan

Yoshiaki Kikuchi

Japan

Yeon-Kyu Kim

Japan

Shingo Kitamura

Japan

Hiromitsu Kobayashi

Japan

Shunsaku Koga

Japan

Satoru Kojima

Japan

Takaaki Kondo

Japan

Katsuyasu Kouda

Japan

Tomoaki Kozaki

Japan

Susumu Kudo

Japan

Soomin Lee

Japan

Akira Maeda

Japan

Takafumi Maeda

Japan

Yoshifumi Miyazaki

Japan

Jordan Moon

United States of America

Takeshi Morita

Japan

Satoshi Muraki

Japan

Yumiko Nagai

Japan

Harunobu Nakamura

Japan

Masashi Nakamura

Japan

Kyuichi Niizeki

Japan

Jun-Ya Ohashi

Japan

Kazue Okamoto-Mizuno

Japan

Anna Ooue

Japan

Melany Richter

Germany

Diane Riddiford-Harland

Australia

Kiyoshi Sanada

Japan

Yayoi Satsumoto

Japan

Kenichi Shibuya

Japan

Yoshihiro Shimomura

Japan

Won Sop Shin

Korea, South

Nami Someya

Japan

Yoshika Takahashi

Japan

Motoharu Takao

Japan

Yuji Takasaki

Japan

Nigel Taylor

Australia

Kumpei Tokuyama

Japan

Sei-Ichi Tsujimura

Japan

Kazuyo Tsuzuki

Japan

Hitoshi Wakabayashi

Japan

Jim Waterhouse

United Kingdom

